# Transcriptome combined single-cell sequencing explores molecular mechanisms of ANGPTL4 in sepsis-induced acute lung injury

**DOI:** 10.1371/journal.pone.0328551

**Published:** 2025-07-31

**Authors:** Ying Qi, Changqi Zhou, Bing Chen

**Affiliations:** Department of Critical Care Medicine, The Second Hospital of Tianjin Medical University, Tianjin, China; The University of Texas Health Science Center at Houston, UNITED STATES OF AMERICA

## Abstract

**Objective:**

Sepsis-induced acute lung injury (ALI) constitutes a critical clinical syndrome associated with high mortality rates, yet its molecular mechanisms remain inadequately elucidated. Recent evidence indicates that ANGPTL4 may influence inflammatory responses and endothelial barrier integrity; however, its cell-specific regulatory mechanisms in sepsis-associated ALI are not well understood. This study utilizes transcriptome profiling combined with single-cell sequencing to systematically analyze the spatiotemporal expression patterns and functional networks of ANGPTL4 during the progression of ALI.

**Methods:**

Gene expression profiles from acute lung injury patients were obtained from the Gene Expression Omnibus (GEO) database. Single-cell and intercellular communication analyses identified candidate gene sets. GSEA examined gene-immune cell relationships, while gene enrichment analysis explored key gene mechanisms. miRNA networks identified target miRNAs for these key genes. Molecular docking with AutoDock and the CTD database predicted drugs interacting with ANGPTL4. Additionally, in vitro experiments confirmed the Angptl4 gene expression level in sepsis-induced acute lung injury.

**Results:**

Angptl4 is a crucial marker for acute lung injury progression, potentially affecting pathways like the pentose phosphate pathway, fatty acid degradation, and PPAR signaling. It may interact with Q9BY76-Quercetin, but this requires further investigation. In vitro studies show a notable increase in Angptl4 expression compared to controls.

**Conclusion:**

The increased expression of ANGPTL4 may influence disease progression through mechanisms involving fatty acid metabolism, PPAR signaling, and the pentose phosphate pathway in murine models. Furthermore, its dual role in regulating inflammation through interactions with both pro-inflammatory and anti-inflammatory cells underscores its pivotal contribution to the pathogenesis of acute lung injury (ALI), thereby supporting the development of targeted therapies for sepsis-induced lung injury.

## Introduction

Sepsis-induced acute lung injury (ALI) is a common complication of sepsis and a significant contributor to morbidity and mortality [1]. Epidemiological studies indicate that ALI affects 30−50% of sepsis patients, with acute respiratory distress syndrome (ARDS) being the most severe form of this condition [2]. The pathophysiology of sepsis-associated ALI is complex, involving the activation of pathogen-associated molecular patterns (PAMPs) and damage-associated molecular patterns (DAMPs), which stimulate Toll-like receptors (TLRs) [3,4]. This activation triggers signaling pathways such as NF-κB, leading to the release of proinflammatory cytokines including TNF-α, IL-1β, and IL-6. Consequently, proteases like elastase and reactive oxygen species (ROS) are released, causing damage to alveolar epithelial cells and the endothelial barrier [5]. These events result in an uncontrolled inflammatory response, endothelial dysfunction, and increased microvascular permeability [6]. Notably, an effective treatment for sepsis-induced acute lung injury remains elusive [7]. Therefore, early identification of high-risk patients and optimization of treatment strategies are essential for improving patient outcomes [8].

ANGPTL4, a member of the angiopoietin family, plays complex roles in lipid metabolism by inhibiting LPL, which affects diabetes and metabolic disorders [9]. Its clinical significance is underscored by studies linking plasma ANGPTL4 levels to sepsis-related lung injury, where it may reduce inflammation [10]. Recent investigations have demonstrated that ANGPTL4 is involved in the modulation of inflammatory signaling and immune responses [11]. ANGPTL4 may affects VEGF-induced capillary formation and integrity,the protein ZO-1, a component of tight junctions, links occludins and claudins to actin in the cytoskeleton; its loss increases intestinal permeability [12,13]. However, it remains uncertain if Angptl4 affects vascular permeability and tight junction proteins in sepsis-related lung injury.the exact mechanisms and pathways involved remain unclear. Animal models of sepsis and mechanical ventilation-induced acute lung injury are being used to investigate ANGPTL4’s role, but its precise impact on acute lung injury development is still not fully understood [14].

We utilized RNA-seq and bioinformatics to study Angptl4 expression and identify dysregulated genes in a mouse model of sepsis-induced acute lung injury. Advances in high-throughput single-cell transcriptome sequencing have enabled the identification of differentially expressed genes, aiding in disease mechanism understanding, molecular typing, and therapeutic target development [15]. Single-cell RNA-sequencing (scRNA-seq) allows for comprehensive transcriptome profiling of thousands of individual cells, facilitating the detection of disease-associated genes. bioinformatics analysis identifies key differentially expressed genes essential for understanding disease pathogenesis [16].

## Materials and methods

### Data download

The Gene Expression Omnibus (GEO) at NCBI provides access to the GSE18341 series matrix file and its annotation file GPL1261. This file contains gene expression data for 30 acute lung injury cases, with 22 in the disease group and 8 in the control group. All samples are from lung tissues, with the original study approved by the Research Committee of the Veterans Affairs Puget Sound Health Care System. Additionally, we accessed the single-cell data file for GSE207651 from the NCBI GEO database, along with two sample data files containing detailed single-cell expression profiles, used for single-cell analysis, comprising one control and one disease case.The raw datasets underlying the experimental and analytical results are provided in S1 Data.

### Ethical approval

The study utilized publicly available datasets from the GEO database that had received ethical approval for data collection. This study followed ARRIVE guidelines and received approval from The Second Hospital of Tianjin Medical University Animal Ethics Committee [Approval number:KY2024K311]. Experiments complied with NIH guidelines for laboratory animal care.

### Immune cell infiltration analysis

The CIBERSORT methodology is a prevalent analytical approach employed for the identification of immune cell types within microenvironments [17]. This technique utilizes support vector regression and deconvolution analysis applied to the expression matrix of immune cell subtypes, incorporating 511 biomarkers and 25 mouse immune cell phenotypes. In our study, we employed the CIBERSORT algorithm to analyze the sample data, enabling us to ascertain the relative proportions of 25 immune cell types and to conduct a correlation analysis between gene expression and immune cell content.

### GSVA analysis

The Gene Set Variation Analysis (GSVA) represents a non-parametric, unsupervised approach for assessing transcriptome-wide gene set enrichment. This method converts gene-level expression changes into pathway-level changes by comprehensively scoring of the gene set of interest, enabling the determination of the biological functions associated with the samples under investigation. In this study, gene sets will be obtained from the Molecular Signatures Database. and the GSVA algorithm will be employed to comprehensively score each gene set, facilitating the evaluating of potential biological function changes across different samples.

### GSEA analysis

The study population was stratified into high and low Angptl4 expression cohorts. Subsequent gene set enrichment analysis (GSEA) was employed to elucidate the differential signaling pathways between these expression groups. The background gene set utilized for this analysis was the version 7.0 annotation gene set, obtained from the MsigDB database as the subtype pathway annotation. The differential expression analysis of the pathway between subtypes was conducted. gene sets with significant enrichment (adjusted p-value < 0.05) were ranked according to the consistency score.

### Regulatory network analysis of important genes

Cistrome DB is a comprehensive resource for analyzing ChIP-seq and DNase-seq, encompassing 58 mouse transcription factors, histone modifications, and chromatin accessibility samples. This study utilized Cistrome DB to explore transcription factor interactions with key genes, configuring the genome file to motif, setting the transcription start site to 10kb, and using Cytoscape for data visualization.

### Construction of miRNA network

MicroRNAs (miRNAs) are small non-coding RNAs that regulate gene expression by degrading mRNA or blocking its translation. This study sought to identify miRNAs linked to key genes involved in harmful gene transcription or degradation [18]. Relevant miRNAs were sourced from the miRDB database, and key gene networks were visualized with Cytoscape software.

### Co-expression analysis

This study examined the co-expression patterns of the Angptl4 gene in sepsis-induced acute lung injury. Using a correlation coefficient threshold of 0.9 and a p-value cutoff of 0.05, we identified the most significantly co-expressed genes with Angptl4. We employed the “WGCNA” and “pheatmap” R packages to create a correlation network and heatmap to illustrate these relationships [19].

### Single-cell analysis

The data were processed using the Seurat package, and the spatial relationships between cell clusters were analyzed using the UMAP algorithm. cell types and their associated marker genes were identified by integrating information from the CellMarker database and a comprehensive review of the literature, enabling robust cell annotation.

### Drug prediction and molecular docking

The Comparative Toxicogenomics Database (CTD) compiles extensive interaction data among chemicals, genes, functional phenotypes, and diseases, serving as a crucial tool for investigating environmental exposure factors and potential drug mechanisms [20]. To forecast potential drugs or molecular compounds interacting with key genes, the CTD was queried. This database encompasses over 46.64 million interactions, including more than 2.3 million chemicals, 46,689 genes, 4,340 phenotypes, and 7,212 diseases.The 3D protein structures corresponding to key genes were retrieved from the AlphaFold database (https://alphafold.com/). Leveraging the CTD database (https://www.ctdbase.org/), key gene-drug predictions were conducted. Relevant compound structures were then accessed via the PubChem database (https://pubchem.ncbi.nlm.nih.gov/). Molecular docking was executed using AutoDock software and a genetic algorithm, with fifty simulations performed. The configuration with the lowest binding energy was visualized in PyMOL to illustrate small molecule and protein binding sites.

### lipopolysaccharide (LPS)-induced ALI in mice

All the animal procedures conducted in the present study conformed to the guidelines of the Care and Use of Laboratory Animals and approved by the Animal Use Committees of The Second Hospital of Tianjin Medical University. Male, healthy, wild-type (WT) C57BL/6J mice (8 weeks old, specific pathogen free) were purchased from the Laboratory Animal Center of Chinese Academy of Medical Sciences (CAMS). Twelve male mice were randomly divided into two groups,Intraperitoneal LPS injection (10 mg/kg) was employed to establish sepsis-induced ALI model according to previous study [21].Mice were randomly divided into two groups: Control group and ALI group. After stimulating with LPS 24 h, animals were sacrificed. Angptl4 mRNA in lung tissues were measured by Real-Time Quantitative PCR (RT-qPCR). The expressions of β-actin, Angptl4 in the lung were detected by Western blot.

### RNA extraction and real-time quantitative PCR assay

Total RNA was extracted from tissues or cells using the RNA/Protein Isolation Kit (Beyotime) following the manufacturer’s instructions. cDNA synthesis was performed with the miRNA All-In-One cDNA Synthesis Kit (ABM), and RT-qPCR was conducted using BlasTaqTM 2X PCR MasterMix (ABM) on a 7500 Real-Time PCR Instrument (Applied Biosystems by Thermo Fisher Scientific). mRNA expression levels were normalized to β-actin, and the threshold cycle (Ct) for all genes was detected to determine relative expression levels. Experiments were conducted in triplicate.

### Western blotting

Lung tissues were homogenized in RIPA buffer with protease inhibitors and sonicated for 15 seconds. Protein concentration was determined using a BCA Protein Assay kit. After centrifugation at 12,000 × g for 15 minutes at 4 °C, proteins were separated by 10% SDS-PAGE and transferred to PVDF membranes. Non-specific binding was blocked with 5% skimmed milk for 60 minutes, followed by overnight incubation at 4 °C with primary antibodies (Angptl4 and β-actin, both at 1:1000). After three washes with TBST, membranes were incubated at 37 °C for 1 hour with anti-rabbit-HRP (1:50000) or anti-mouse-HRP (1:10000). Protein expression was detected using enhanced chemiluminescence, and grey analysis was performed using ImageJ software.

### Statistical analysis

All statistical analyses were performed using R (version 4.3.0), with p < 0.05 considered statistically significant. Data are presented as means ± standard deviation (SD). paired t-tests were employed to determine statistical significance between groups, using GraphPad Prism 9. A threshold of p < 0.05 was adopted for statistical significance.

## Results

### Differential Angptl4 expression and ROC curve analysis

**Fig 1:**
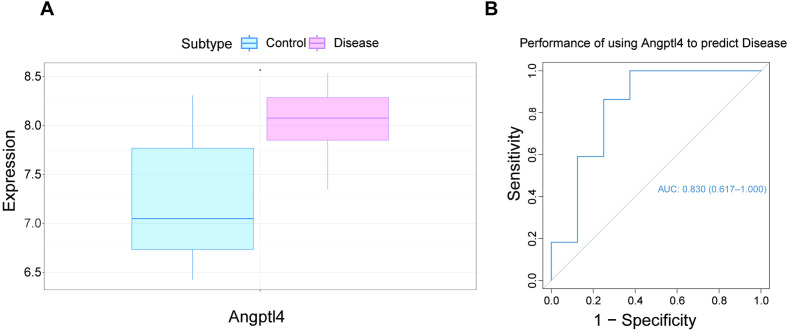
Differential Angptl4 expression and ROC curve analysis.

The dataset GSE18341 was obtained from the NCBI GEO public repository, encompassing a control group (n = 8) and a disease group (n = 22). Differential expression analysis revealed a significant upregulation of Angptl4 in the disease group compared to the control (Fig 1A). The predictive performance of Angptl4 was further evaluated through receiver operating characteristic (ROC) curve analysis. The area under the curve (AUC) for Angptl4 was 0.830 (95% CI: 0.617–1.000), suggesting its strong predictive potential for disease occurrence and progression (Fig 1B).

### Angptl4 levels correlate with immune infiltration in disease

**Fig 2:**
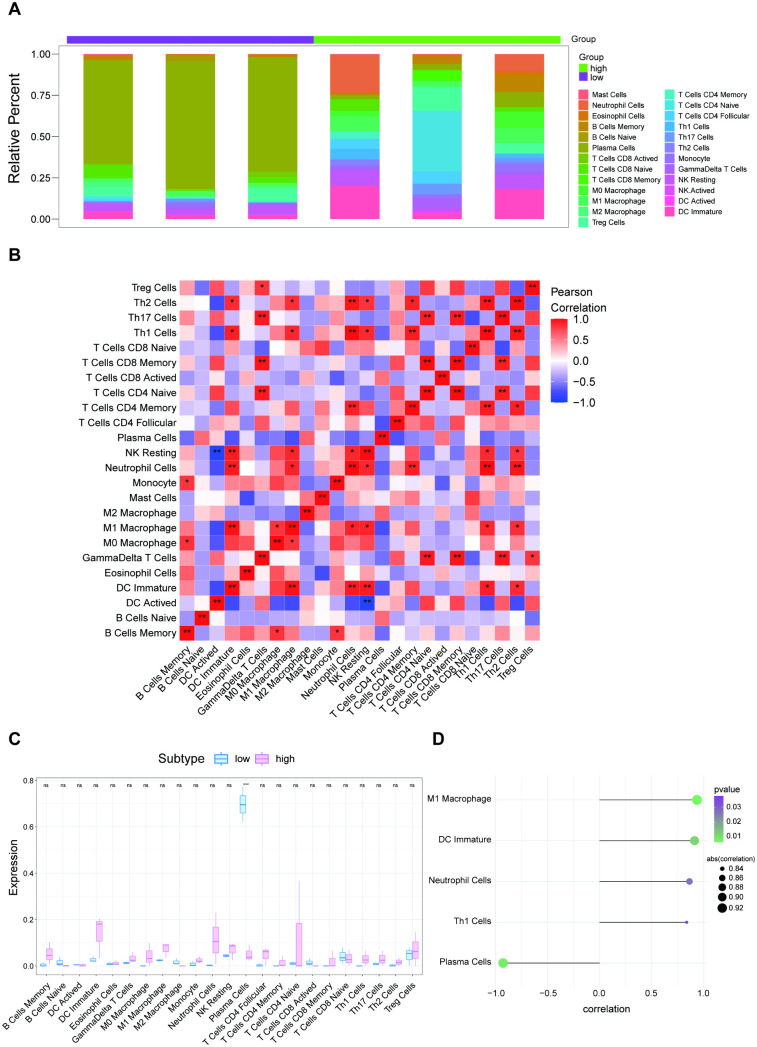
Immune infiltration profiles, plasma cell differences, and Angptl4 correlations with M1 Macrophages, DC Immature, and Plasma Cells.

**Fig 3:**
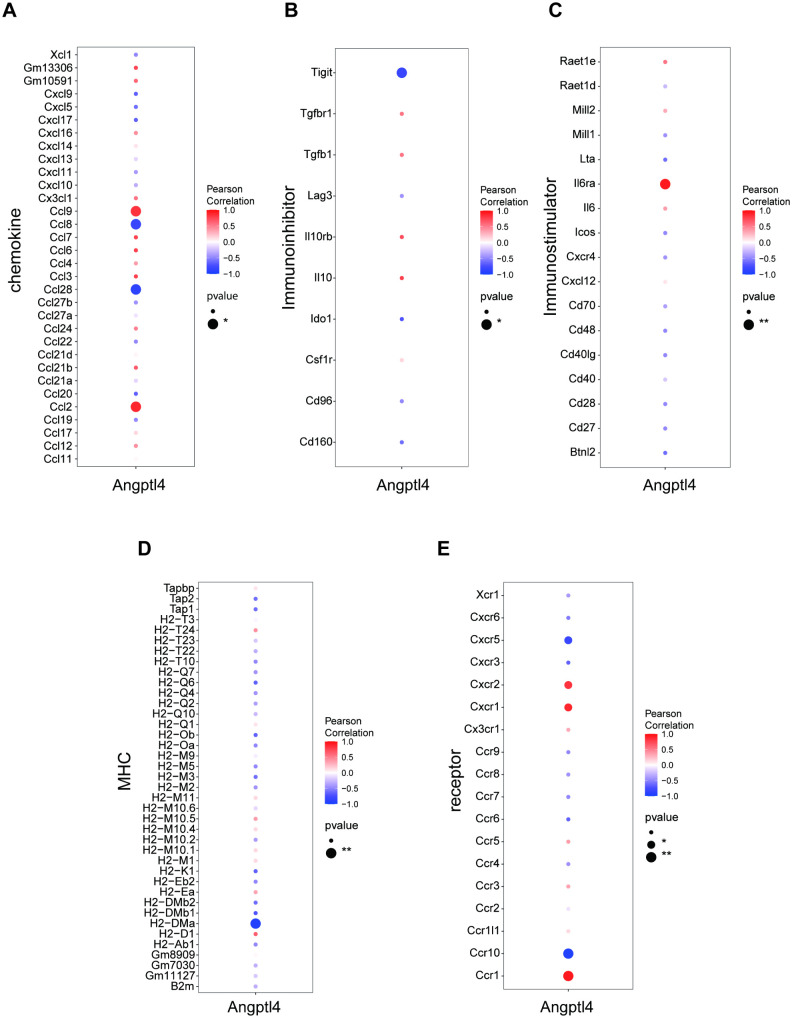
Correlation of Angptl4 with immunomodulatory factors, chemokines, and cell receptors from the TISIDB database.

The study examines the link between Angptl4 expression and immune infiltration in diseases, highlighting differences in Plasma Cells between groups (Fig 2A–2C). The findings revealed a substantial positive correlation between Angptl4 and M1 Macrophages, DC Immature cells, among others, and a notable negative correlation with Plasma Cells (Fig 2D). Using TISIDB data, this study explored the correlations of the Angptl4 gene with chemokines, immunoinhibitors, immunostimulators, MHC, and receptors (Fig 3). These analyses indicate a close link between Angptl4 and the extent of immune cell infiltration and the immune microenvironment.

### Gene set enrichment analysis and gene set variation analysis

**Fig 4:**
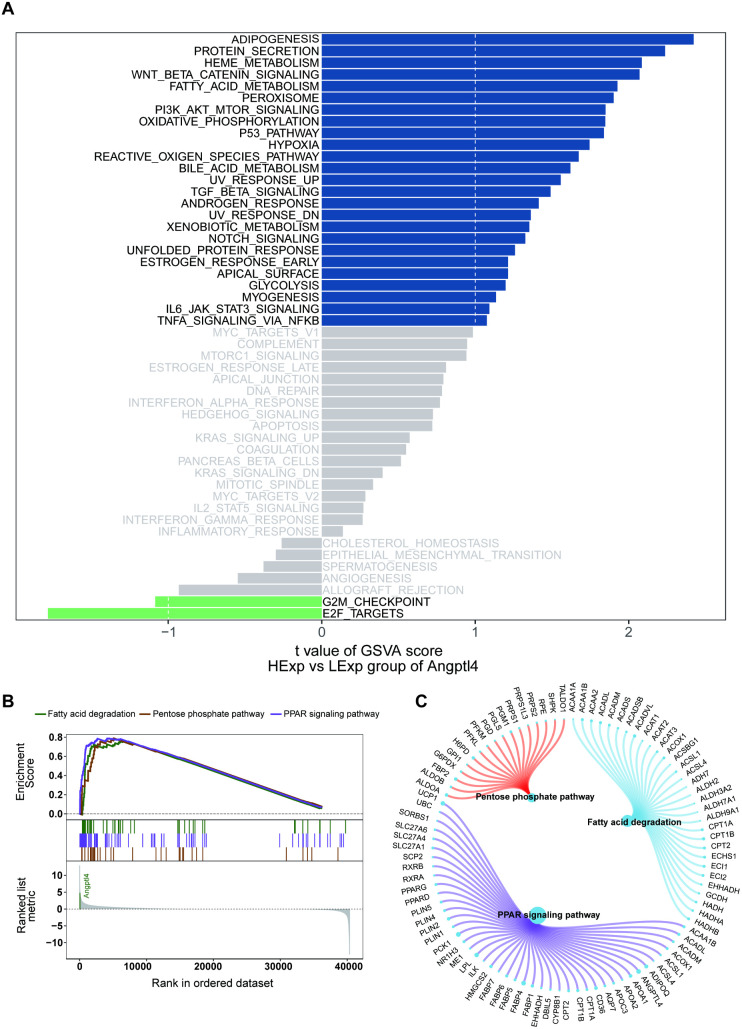
Angptl4 correlates with key metabolic pathways, including adipogenesis and PPAR signaling enhancement.

Gene Set Enrichment Analysis (GSEA) was conducted using the MSigDB dataset (https://www.gsea-msigdb.org/gsea/msigdb/index.jsp), and the enrichment analysis results were visualized using the ggplot2 package. The GSVA findings indicate that higher Angptl4 expression is associated with enriched pathways like ADIPOGENESIS, PROTEIN SECRETION, HEME METABOLISM, and FATTY ACID METABOLISM (Fig 4A). Additionally, GSEA results suggest Angptl4 may enrich pathways such as the Pentose phosphate pathway, Fatty acid degradation, and PPAR signaling pathway (Fig 4B and 4C). hereby implying a potential role of Angptl4 in disease progression through these pathways.

### The transcriptional regulatory network involving Angptl4

**Fig 5:**
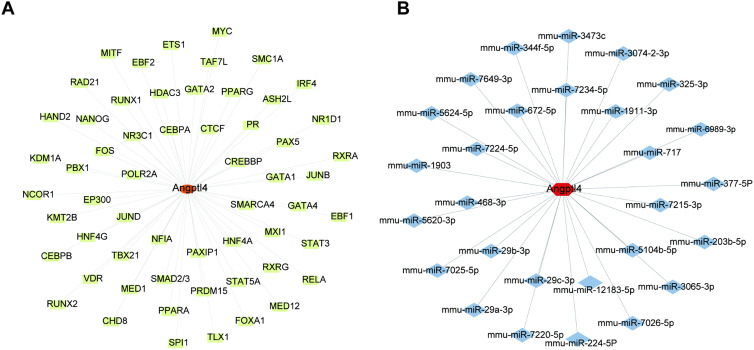
Angptl4 regulatory network in sepsis-induced lung injury and key genes predicted via miRDB.

The study used the Angptl4 gene set to explore its transcriptional regulatory network. By utilizing the Cistrome DB database, 58 potential transcription factors for Angptl4 were identified and visualized with Cytoscape to create a regulatory network related to sepsis-induced acute lung injury. (Fig 5A). Additionally, the miRDB database helped identify 28 miRNAs and their 28 interaction pairs with mRNA, which were also visualized using Cytoscape (Fig 5B).

### Co-expression analysis

**Fig 6:**
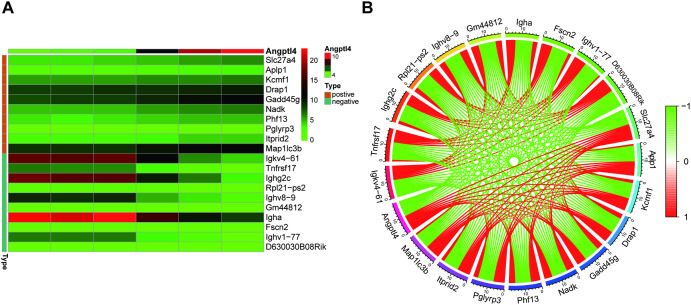
Heat map of top 10 correlated genes and co-expression circle map.

Genes exhibiting significant co-expression with Angptl4 were identified, and heatmap visualizations were generated to depict the top 10 genes displaying both positive and negative correlations (Fig 6A).Furthermore, a circular plot was constructed to illustrate the gene co-expression correlations (Fig 6B).

### Single-cell analysis

**Fig 7:**
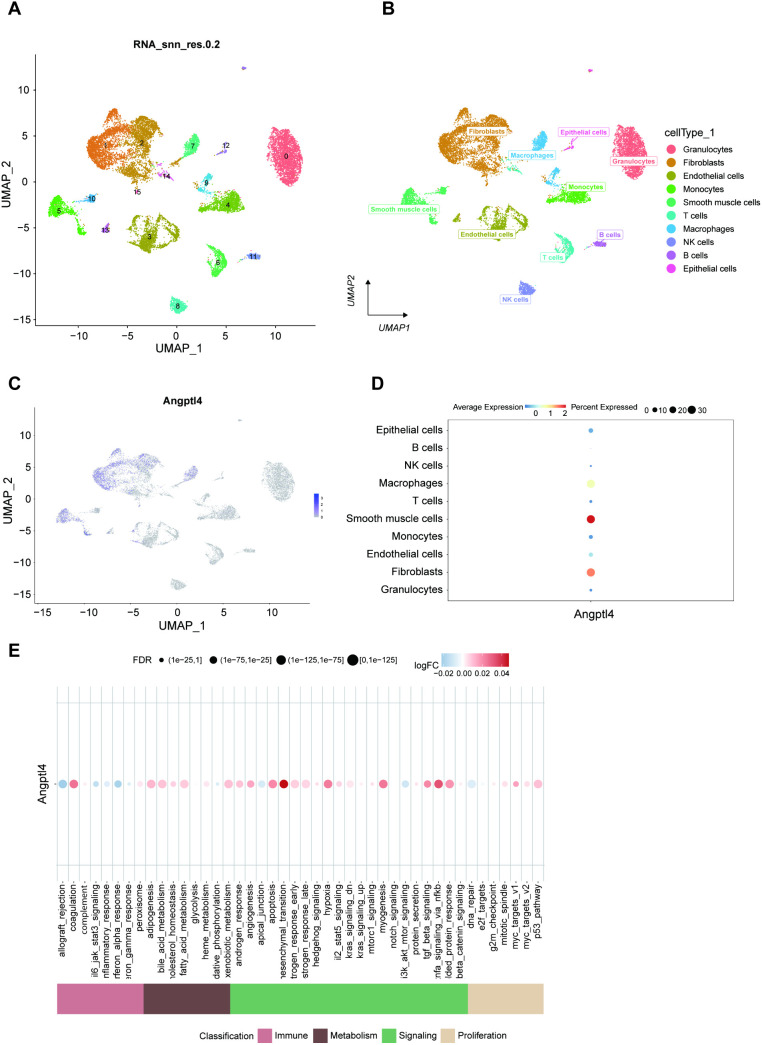
Single-cell analysis identifies 10 cell classes and Angptl4 expression in key signaling pathways.

The single-cell data file of GSE207651 was downloaded from the NCBI GEO public database, Cell clustering via the UMAP algorithm identified 16 subtypes (Fig 7A). which were subsequently categorized into 10 cell types: Granulocytes, Fibroblasts, Endothelial cells, Monocytes, Smooth muscle cells, T cells, Macrophages, NK cells, B cells, and Epithelial cells (Fig 7B). The following investigation will explore the expression of Angptl4 across these specific cell types (Fig 7C and 7D). Additionally, the AUCell function quantified immune and metabolic pathway levels within single-cell data, and a bubble chart illustrated Angptl4’s activity differences in pathways related to immune metabolism. The findings revealed heightened Angptl4 activity in the coagulation, epithelial-mesenchymal transition, myogenesis, and TNF-α signaling via NF-κB pathways (Fig 7E).

### Drug prediction and molecular docking

**Fig 8:**
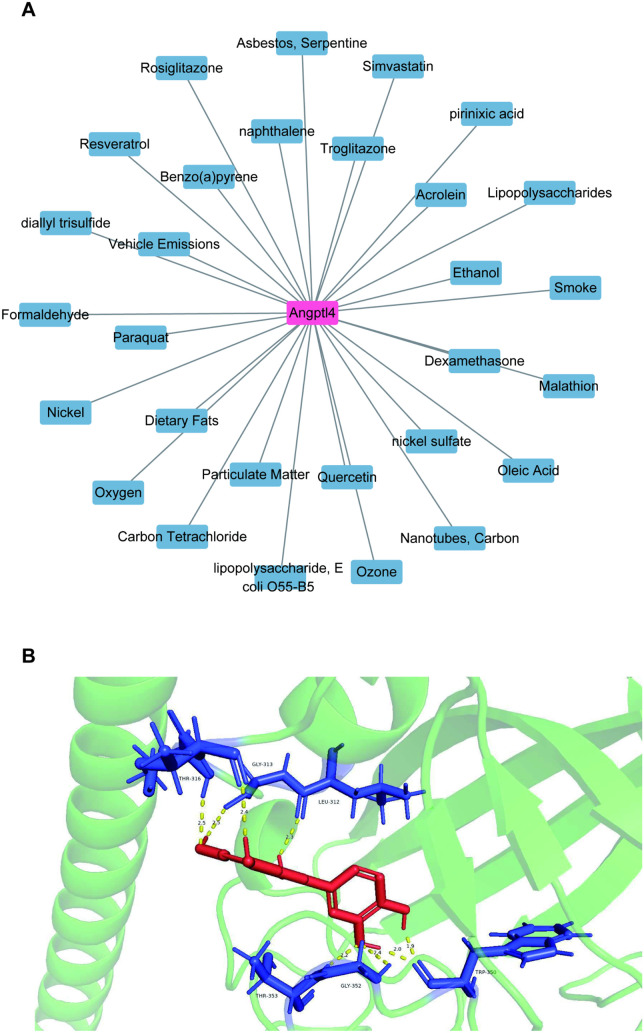
Quercetin is a key gene that interacts with Angptl4.

This study utilized the CTD database to identify drugs potentially interacting with key genes, revealing 29 drugs associated with Angptl4 (Fig 8A). Molecular docking was subsequently conducted, focusing on the interaction between the protein ANGPTL4:Q9BY76 and the compound Quercetin. Molecular docking showed that Quercetin binds to ANGPTL4:Q9BY76 with an energy of −5.28 kcal/mol (Fig 8B).

### Validation of Angptl4 expression in ALI

**Fig 9:**
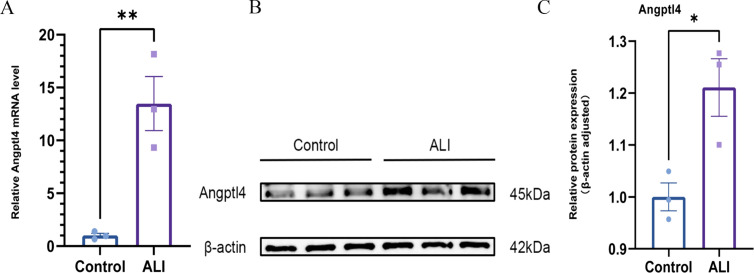
Validation of Angptl4 by qPCR and Western blot.

In this study, we established an acute lung injury (ALI) model in C57BL/6J mice using lipopolysaccharide, dividing them into control and model groups (n = 6 per group). qPCR analysis demonstrated that the mRNA expression of Angptl4 in the lung tissue of ALI model mice was significantly higher than that in the control group (p < 0.05), showing an increased relative expression level. Western blot analysis further verified these changes at the protein level, revealing that the protein expression of Angptl4 in the lung tissue of the ALI group was significantly upregulated compared to the control group (p < 0.05) (S1 Fig). Representative western blot images showing Angptl4 and β-actin protein expression are provided in S1 Fig. The list of chemicals, reagents, and primers used in this study is provided in S1 Table. All data were statistically analyzed using GraphPad Prism 9, with significant differences noted (Fig 9).

## Discussion

Sepsis-induced acute lung injury (ALI) and acute respiratory distress syndrome (ARDS) involve the disruption of the pulmonary microvascular endothelial barrier, leading to pathological vascular hyperpermeability and life-threatening pulmonary oedema [22,23]. Clinically, sepsis mortality rates range from 20% to 30%, increasing to 40%−60% with ALI/ARDS complications and surpassing 50% in severe ARDS cases. Common symptoms include shortness of breath, chest pain, and respiratory failure [24]. Managing sepsis-induced acute lung injury (ALI) focuses on supportive care, infection control, and enhanced oxygenation, with early diagnosis and treatment being crucial for effective management.

Angiopoietin-like protein 4 (Angptl4), a multifunctional glycoprotein of the angiopoietin-like family, has emerged as a crucial regulator of endothelial integrity [25]. Clinical studies have found that lower serum Angptl4 levels in sepsis patients correlate with increased disease severity and lung injury, suggesting its potential as an early diagnostic or prognostic biomarker [26]. Previous investigations have connected ANGPTL4 activity with the maintenance of endothelial cell integrity, as well as with inflammation, oxidative stress, and lipid metabolism [27,28].Recent advancements underscore Angptl4’s dual function in maintaining endothelial barrier integrity by stabilizing intercellular junction proteins like VE-cadherin, and in modulating systemic inflammation by inhibiting TNF-α/IL-6 signaling and NF-κB pathways [29]. These diverse mechanisms position Angptl4 as a promising therapeutic candidate for addressing both the vascular and inflammatory-metabolic components of sepsis-associated lung injury [30]. Angptl4 is essential for boosting tight junction proteins like ZO-1 and occludin, reducing endothelial gaps, and counteracting VEGF-induced vascular permeability [31].

In this study, we sought to elucidate the role of Angptl4 in sepsis-associated acute lung injury (ALI) and to systematically elucidate its synergistic effect through multiple mechanisms, including anti-inflammation, endothelial barrier stabilization, metabolic regulation, and antioxidants. This multi-target mode of action has the potential to overcome the limitations of traditional single-mechanism drugs and provides a theoretical basis for the development of comprehensive intervention strategies [32].

This study makes significant contributions by establishing a new link between Angptl4’s metabolic regulation and the immune-inflammatory response, thereby unveiling the complex interplay between metabolic disorders and organ damage in sepsis. It lays the groundwork for developing metabolic-immunological combination therapies [33]. The innovation of the study lies in its integration of multidisciplinary tools, including molecular biology, metabolomics, and gene editing technology, which systematically analyze the central role of Angptl4 from the mechanistic to the application level, and propose a viable translational pathway [34].

Combining transcriptome and single-cell sequencing offers a detailed understanding of molecular mechanisms in diseases like sepsis-induced acute lung injury (ALI) [35]. Single-cell RNA sequencing (scRNA-seq) allows researchers to explore the lung’s cellular landscape in ALI, identifying cell types and pathways affected by ANGPTL4. This integrated approach enhances insights into molecular pathways and cellular interactions in ALI, clarifying the role of proteins like ANGPTL4 and revealing dynamic changes in the lung microenvironment during injury and recovery.

This study examined the link between Angptl4 expression and immune infiltration in acute lung injury (ALI), noting differences in plasma cell infiltration among groups. Angptl4 showed a positive correlation with pro-inflammatory immune cells like M1 macrophages and immature dendritic cells, but a negative one with plasma cells [1]. TISIDB data further revealed strong associations between Angptl4 and various immunoregulatory factors, including chemokines, immunoinhibitors, immunostimulators, MHC molecules, and immune receptors. These results suggest Angptl4 influences the immune environment in ALI by affecting immune cell recruitment and activation [36]. The imbalance in plasma cell infiltration and M1 macrophage polarization indicates Angptl4’s dual role in managing inflammation and tissue repair [37]. Future research should focus on understanding how Angptl4 regulates these pathways, potentially offering therapeutic targets for reducing immune-related lung injury.

Molecular docking with Autodock Vina suggests interactions between ANGPTL4 and quercetin, but the exact pathways of ANGPTL4’s effects are unknown, highlighting a research gap [38]. Further studies are needed on the PPAR isoforms (PPARα, PPARβ/δ, PPARγ) linked to ANGPTL4’s role in regulating endothelial barrier integrity, especially regarding tight junction proteins like ZO-1 and occludin [39]. Quercetin’s therapeutic potential is being recognized, as it reduces cigarette smoke-induced lung injury by inhibiting inflammation and lessens myocardial ischemia/reperfusion injury in rats via the PI3K/Akt pathway [40]. Research should focus on the ANGPTL4-PPAR axis’s mechanisms and quercetin’s efficacy in treating barrier dysfunction in inflammatory diseases.

Future research should focus on precisely elucidating the molecular mechanisms of Angptl4 and investigating its clinical applications in the treatment of sepsis and acute lung injury (ALI). The present study is subject to certain limitations: Angptl4’s effects may vary across different tissues, potentially complicating therapeutic interventions; its specific molecular pathways in inflammation, endothelial function, and metabolism remain ambiguous; and the association between serum Angptl4 levels and disease severity requires confirmation in larger and more diverse cohorts [41]. Our qPCR and Western blot analyses in an acute lung injury (ALI) animal model showed a significant increase in Angptl4 mRNA and protein in lung tissues compared to controls, highlighting its tissue-specific role. This raises the question of whether elevated Angptl4 in ALI is protective or harmful, as its effects may depend on the inflammatory context and metabolic needs. These uncertainties underscore the need for interdisciplinary research to develop Angptl4-based therapies. Future studies should explore small molecule agonists or CRISPR-based gene editing targeting Angptl4, while considering its tissue-specific effects, targeted delivery, and potential side effects [42].

## Conclusion

In murine models, elevated ANGPTL4 expression may potentially influence disease progression through mechanisms that could involve fatty acid metabolism, PPAR signaling, and the pentose phosphate pathway. Its interactions with both pro-inflammatory and anti-inflammatory cells highlight its dual function in the regulation of inflammation. These findings underscore the critical role of ANGPTL4 in the pathogenesis of acute lung injury (ALI) and advocate for the development of innovative therapeutic strategies targeting sepsis-associated lung injury.

## Supporting information

S1 Data Raw datasets underlying the experimental and analytical results presented in this study. (XLS)

S1 Fig Representative western blot images demonstrating Angptl4 and β-actin protein expression. (TIF)

S1 Table List of chemicals, reagents, and primers used in this study. (DOCX)
