# Supplementary figures and images for: Transcriptome combined single-cell sequencing explores molecular mechanisms of ANGPTL4 in sepsis-induced acute lung injury
Source: PLoS One. 2025 Jul 31;20(7):e0328551. doi: 10.1371/journal.pone.0328551 (PMC12312960; doi:10.1371/journal.pone.0328551)

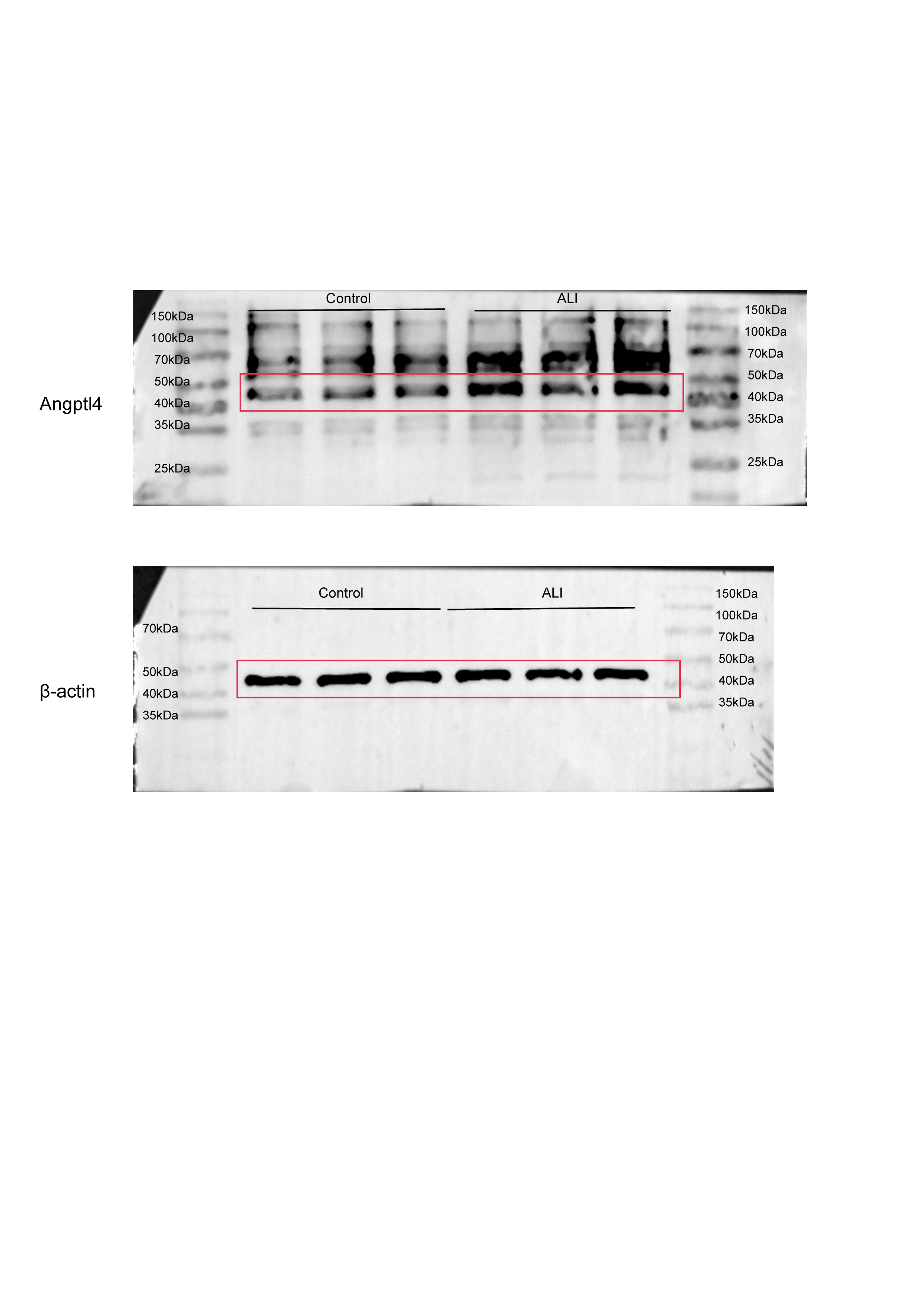

Supplement: S1 Fig — (TIF) [file pone.0328551.s002.TIF]
