# Supplementary material for: Transcriptome combined single-cell sequencing explores molecular mechanisms of ANGPTL4 in sepsis-induced acute lung injury
Source: PLoS One. 2025 Jul 31;20(7):e0328551. doi: 10.1371/journal.pone.0328551 (PMC12312960; doi:10.1371/journal.pone.0328551)
Supplement: S1 Table — (DOCX) [file pone.0328551.s003.docx]

**Supplementary Table 1. Details of Chemical and Reagents**

| **REAGENT or RESOURCE** | **SOURCE** | **IDENTIFIER** |
| --- | --- | --- |
| Anti-β-Actin Mouse Monoclonal Antibody (1C7) | Abbkine | Cat# ABL1010 |
| Anti-ANGPTL4 | Abcam | Cat# ab196746 |
| HRP, Goat Anti-Mouse IgG | Abbkine | Cat# A21010 |
| HRP, Goat Anti-Rabbit IgG | Abbkine | Cat# A21020 |
| SuperKine™ West Femto Maximum Sensitivity Substrate | Abbkine | Cat# BMU102-CN |
| uperKine™ Enhanced Antibody Dilution Buffer | Abbkine | Cat# BMU103-CN |
| Omni-Easy™ One-step Color PAGE Gel Rapid Preparation Kit（10%） | Epizyme | Cat# PG212 |
| Multicolor Prestained Protein Ladder | Epizyme | Cat# WJ103 |
| Tris-Glycine-SDS | Epizyme | Cat# TF101 |
| Ice bath free rapid transfer buffer instant granules (wet transfer method) | Epizyme | Cat# TF105 |
| Skimmed Milk Powder | Epizyme | Cat# PS112 |
| miRNA All-In-One cDNA Synthesis Kit | ABM | Cat# G898 |
| BlasTaqTM 2X PCR MasterMix | ABM | Cat# G895 |
| PVDF | Beyotime | Cat# FFP32 |
| lipopolysaccharide（O111:B4） | Beyotime | Cat# S1732-5mg |
| Enhanced BCA Protein Assay Kit) | Beyotime | Cat# P0010 |
| RNA/Protein Isolation Kit | Beyotime | Cat# R0018M |
| Eppendorf Mastercycler | Eppendorf | Cat#Mastercycler Personal |
| Tribromoethanol for ready use | Meilunbio | Cat#MA0478 |
| Mini-PROTEAN® Tetra | Bio-Rad | Cat#1658001 |
| Chemiluminescent and fluorescent imaging systems | Clinx | Cat#ChemiScope 6100 |
| 7500 Real-Time PCR Instrument | Applied Biosystems byThermo Fisher Scientific | Cat# 7500 |

**Supplementary Table 2. List of qPCR primers**

| **Gene** | **Forward** | **Reverse** |
| --- | --- | --- |
| Mouse-β-actin | CACTGTCGAGTCGCGTCC | TCATCCATGGCGAACTGGTG |
| Mouse-Angptl4 | CTGGACAGTGATTCAGAGACGC | GATGCTGTGCATCTTTTCCAGGC |
